# Poly-(ADP-ribose) polymerases inhibition by olaparib attenuates activities of the NLRP3 inflammasome and of NF-κB in THP-1 monocytes

**DOI:** 10.1371/journal.pone.0295837

**Published:** 2024-02-09

**Authors:** Khamis Mustafa, Ying Han, Dan He, Ying Wang, Nan Niu, Pedro A. Jose, Yinong Jiang, Jeffrey B. Kopp, Hewang Lee, Peng Qu

**Affiliations:** 1 Institute of Heart and Vessel Diseases, The Second Affiliated Hospital, Dalian Medical University, Dalian, China; 2 Department of Cardiology, Jinqiu Hospital of Liaoning Province, Shenyang, China; 3 Department of Cardiology, The Second Affiliated Hospital, Dalian Medical University, Dalian, China; 4 Department of Medicine, The George Washington University School of Medicine & Health Sciences, Washington, District of Columbia, United States of America; 5 Department of Physiology/Pharmacology, The George Washington University School of Medicine & Health Sciences, Washington, District of Columbia, United States of America; 6 Department of Cardiology, The First Affiliated Hospital, Dalian Medical University, Dalian, China; 7 Kidney Disease Section, Kidney Diseases Branch, National Institute of Diabetes and Digestive and Kidney Diseases, National Institutes of Health, Bethesda, Maryland, United States of America; 8 Faculty of Medicine, Dalian University of Technology, Dalian, China; Guangdong Medical University, CHINA

## Abstract

Poly-(ADP-ribose) polymerases (PARPs) are a protein family that make ADP-ribose modifications on target genes and proteins. PARP family members contribute to the pathogenesis of chronic inflammatory diseases, including atherosclerosis, in which monocytes/macrophages play important roles. PARP inhibition is protective against atherosclerosis. However, the mechanisms by which PARP inhibition exerts this beneficial effect are not well understood. Here we show that in THP-1 monocytes, inhibition of PARP by olaparib attenuated oxidized low-density lipoprotein (oxLDL)-induced protein expressions of nucleotide-binding oligomerization domain, leucine-rich repeat and pyrin domain-containing-3 (NLRP3) inflammasome components: NLRP3, apoptosis-associated speck-like protein containing a caspase activation and recruitment domain (ASC), and caspase-1. Consistent with this effect, olaparib decreased oxLDL-enhanced interleukin (IL)-1β and IL-18 protein expression. Olaparib also decreased the oxLDL-mediated increase in mitochondrial reactive oxygen species. Similar to the effects of the NLRP3 inhibitor, MCC950, olaparib attenuated oxLDL-induced adhesion of monocytes to cultured human umbilical vein endothelial cells and reduced foam cell formation. Furthermore, olaparib attenuated the oxLDL-mediated activation of nuclear factor (NF)-κB through the oxLDL-mediated increase in IκBα phosphorylation and assembly of NF-κB subunits, demonstrated by co-immunoprecipitation of IκBα with RelA/p50 and RelB/p52 subunits. Moreover, PARP inhibition decreased oxLDL-mediated protein expression of a NF-κB target gene, *VCAM1*, encoding vascular cell adhesion molecule-1. This finding indicates an important role for NF-κB activity in PARP-mediated activation of the NLRP3 inflammasome. Thus, PARP inhibition by olaparib attenuates NF-κB and NLRP3 inflammasome activities, lessening monocyte cell adhesion and macrophage foam cell formation. These inhibitory effects of olaparib on NLRP3 activity potentially protect against atherosclerosis.

## Introduction

Poly ADP-ribosylation, carried out by poly-(ADP-ribose) polymerase (PARP), is a posttranslational modification of proteins involved in a wide variety of cellular activities, including DNA repair, transcriptional regulation, inflammation, tumorigenesis, and cell death [1, 2]. Increased activation of PARP contributes to the pathogenesis of diverse chronic inflammatory diseases, and PARP inhibition shows promise in the treatment of these diseases [3, 4], but its role in atherosclerosis is not well known.

Atherosclerosis is a slowly progressive chronic inflammatory condition [5]. The inflammatory response in atherosclerosis is initiated by damage to the vascular endothelium, followed by monocytes entering the arterial wall; subsequently, other leukocytes are recruited [6]. These cells release diverse vasoactive molecules [6]. Monocytes differentiate into macrophages, which proliferate and ingest oxidized low-density lipoprotein (oxLDL) [7]. These lipids drive monocytes’ differentiation into foam cells, which form plaques [7].

The nucleotide-binding oligomerization domain (NOD), leucine-rich repeats (LRR), and pyrin domain-containing 3 (NLRP3) inflammasome is an intracellular multi-protein complex that consists of three components: 1) NLRP3, 2) the adaptor protein ASC [apoptosis-associated speck-like protein containing a caspase activation and recruitment domain (CARD)], and 3) caspase-1 [8]. This inflammasome is an important contributor to the inflammatory response in monocytes/macrophages and to the development of atherosclerosis [8].

In the innate immune system, danger signals, including crystalline cholesterol, oxLDL, reactive oxygen species (ROS), and mitochondrial dysfunction, trigger inflammatory responses [9]. NLRP3 inflammasome activation leads to the maturation of interleukin (IL)-1β and IL-18, which are the key contributors to the inflammatory reaction in the vascular wall and to atherosclerotic plaque formation [10]. The canonical dual-signal model for the activation of NLRP3 inflammasomes proposes that a priming signal induces the upregulation of NLRP3 and pro-IL-1β/pro-IL-18, via activation of the nuclear factor κ-light-chain-enhancer of activated B cells (NF-κB) and a second signal triggers the assembly of the inflammasome complex [11, 12].

NF-κB is a transcription factor protein complex that plays a central role in the inflammatory response [13]. The NF-κB family has five members: NF-κB1 (p105; p50), NF-κB2 (p100; p52), RelA (p65), RelB, and c-Rel [13, 14]. In the inactive state, NF-κB heterodimers (*e*.*g*., p50 with RelA, RelB, or c-Rel) are sequestered in the cytoplasm by IκB, an inhibitory protein. Activation of NF-κB is initiated by the phosphorylation of IκB by the IκB kinase (IKK), following which the NF-κB heterodimers dissociate from IκB and translocate to the nucleus [14]. Activated NF-κB induces gene expression of a wide range of proinflammatory molecules, including cytokines, chemokines, and others [15].

In this study, we investigated the role of oxLDL in the activation of PARP in THP-1 cells, a human monocytic leukemia cell line. We further explored the anti-inflammatory effects of olaparib, a small molecule PARP inhibitor [16], on NLRP3 inflammasome activity and subsequent modulation of pathophysiological processes, including the secretion of proinflammatory cytokines, cell adhesion, and foam cell formation. We also investigated the effects of olaparib on NF-κB activity in the context of oxidative stress-induced inflammatory responses in THP-1 monocytes.

## Materials and methods

### Reagents and antibodies

MCC950, a small molecule NLPR3 inhibitor, and olaparib were purchased from Selleck Chemicals (Houston, TX); oxLDL was purchased from Anhui Yiyuan Bio-technology (Bozhou, China); HyClone RPMI 1640 and DMEM were purchased from Cytiva (Shanghai, China); fetal bovine serum (FBS) and MitoSox Red were purchased from Thermo Fisher Scientific (Beijing, China); aprotinin and leupeptin were purchased from Solarbio Life Science (Beijing, China); DTT (dithiothreitol) was purchased from Wako Pure Chemical Industries (Osaka, Japan); protein A/G-agarose was purchased from Santa Cruz Biotechnology (Shanghai, China); anti-vascular cell adhesion molecule (VCAM)-1, anti-caspase-1, anti-RelA (phosphor-S536), and anti-vinculin antibodies were purchased from Abcam; anti-IL-1β and anti-IL-18 antibodies were purchased from Sab Biotech (Nanjing, China); anti-NLRP3, anti-RelA and anti-phosphor-IκВα (ser 32) antibodies, and a NF-κВ non-canonical pathway antibody sampler kit including anti-IKKα, anti-RelB and anti-p52 antibodies were purchased from Cell Signaling Technology (Shanghai, China); anti-ASC, anti-IκВα, and anti-p50 antibodies were purchased from Proteintech Group (Wuhan, China). Other reagents, unless otherwise stated, were purchased from Sigma-Aldrich (Shanghai, China).

### Cell culture

Human monocytic THP-1 cells obtained from the American Type Culture Collection (ATCC), Manassas, VA] and were cultured in RPMI 1640 medium, supplemented with 10% heat-inactivated FBS at 37°C in a 5% CO_2_/95% air humidified incubator, as previously described [17]. Human umbilical vein endothelial cells (HUVECs) (ATCC) were cultured in DMEM medium, supplemented with 10% heat-inactivated FBS.

### PARP-1 activity assay

PARP-1 activity was measured using a fluorometric kit (MilliporeSigma, Cat. No. 17–10149) following the manufacturer’s instructions. THP-1 cells were treated with vehicle or oxLDL in the presence or absence of various concentrations of olaparib. Cell lysates were incubated with activated DNA, β-nicotinamide adenine dinucleotide (NAD), and recombinant nicotinamidase at 30°C on a plate shaker with gentle agitation for 30 min. The developing reagent was added to each well under dim light and the cells were incubated with light protection for an additional 30 min at room temperature with gentle agitation on a plate shaker. The fluorescence intensity of ammonia from nicotinamide was read on a fluorometer, with excitation at ~420 nm and emission at ~450 nm (Molecular Devices SpectraMax i3x, San Jose, CA).

### Measurement of mitochondrial ROS

Mitochondrial ROS were quantified by MitoSOX Red (2.5 μM; Invitrogen), using flow cytometry, as previously described [18]. THP-1 cells (2 × 10^6^ cells) were exposed to 5 μM MitoSOX Red and incubated in fresh RPMI 1640 medium, protected from light, at 37°C for 10 min. After washing with pre-warmed RPMI 1640 medium and removing the cell aggregates by filtering through a nylon mesh (40 μm), mitochondrial ROS in THP-1 cells were quantified at wavelengths of 510 nm excitation and 580 nm emission by flow cytometry, using a flow-activated cell sorter (FACS) flow cytometry system (Becton Dickinson, San Jose, CA).

### THP-1 cell adhesion assay

HUVECs (5x10^3^ cells/well) were seeded in matrix-coated 96-well plates and incubated at 37°C, 5% CO_2_ for 24 hr at which time HUVECs reached near 100% confluency. THP-1 cell suspensions (4x10^5^/mL) were exposed to 2’,7’-bis-(2-carboxyethyl)-5-(6)-carboxyfluorescein acetoxymethyl ester (BCECF/AM, 5 μM) for 1 hr. After washing 3 times with ice-cold 1x PBS, the cell pellets were re-suspended with fresh RPMI 1640. The BCECF-labeled THP-1 cells (4 x 10^4^ in 100 μL suspension) were seeded on to confluent HUVEC monolayers and co-cultured in 96-well plates in a humidified incubator (37°C, 5% CO_2_) for 1 hr. OxLDL, olaparib, and MCC950 individually or in combination were added in the culture medium. Nonadherent cells were removed by gentle washing, and the number of adherent THP-1 monocytes was counted with a microscope, using ImageJ software (NIH, Bethesda, MD).

The average value of adhesive THP-1 cells in the vehicle treatment group from four (n = 4) experiments was considered as 100%; the relative adhesion of all other groups was normalized to that of vehicle group.

### Foam cell formation

Foam cells were generated from THP-1 cells on glass slides. THP-1 cells were exposed to phorbol myristate acetate (PMA, 100 ng/mL) for 48 hr, with subsequent oxLDL (100 μg/mL) exposure in the presence or absence of olaparib (5 μM) and/or MCC950 (2 μM). After gentle washing 3 times with ice cold 1x PBS buffer, the cells were stained with 0.2% oil red O (ORO, Sigma-Aldrich) for 10 sec. After de-staining with isopropanol for 15 sec and washing with 1xPBS for three times, the cells were stained with hematoxylin (Sigma-Aldrich) for 30 sec. After a final wash with 1x PBS, the coverslips were mounted on slides, using Permount solution (Fisher, Waltham MA).

Foam cells, identified as red-stained cells, were visualized via light microscopy (Leica Biosystems, USA) with 40×magnification and photographed using a Canon DS126431 digital camera (Tokyo, Japan). The optical density of ORO staining, which was considered to be proportional to the amount of oxLDL taken up by macrophages, was quantified at 520 nm with SpectraMax 190 Microplate Reader (Molecular Devices, San Jose, CA).

The average optical density of ORO in the vehicle treatment group from three (n = 3) experiments was considered as 100%; the relative optical density at 520 nm of all other groups was normalized with the vehicle group.

### Western blotting

Western blotting was performed as previously described [17]. Briefly, cell pellets were harvested and protein concentrations were determined using a BCA Protein Assay Kit (Thermo Fisher Scientific). The cell lysate proteins were separated with SDS-PAGE and transferred onto nitrocellulose membranes (Millipore). The membranes were blocked with 5% nonfat milk and incubated overnight at 4°C with primary antibodies. After incubation with the appropriate secondary antibodies at room temperature for 2 hr, the protein bands were detected using the Odyssey near-infrared imaging system (LI-COR Biosciences, Lincoln, NE).

### Co-immunoprecipitation

To identify selected protein-protein interactions involving the NF-κB pathway, co-immunoprecipitation (Co-IP) experiments were performed, as described [19]. Briefly, cell lysates (100 μg of protein) were incubated (rocking platforms, 4°C, 4 h) with 2 μg of anti-IκBα, anti-p50, anti-p52, anti-RelA, or anti-RelB antibodies in TENDS buffer (20 mM Tris HCl, pH 8.0; 1 mM EDTA; 1 mM NaN_3_; 2 mM DTT; 0.25 M sucrose) with 0.5 mM 4-(2-aminoethyl) benzenesulfonyl fluoride (AEBSF), 0.5 mM benzamidine hydrochloride, and protease inhibitors. After adding 60 μL of a 50% slurry of protein A/G-agarose in 1x PBS and incubation at 4°C overnight, the beads were washed three times with 1 mL of ice-cold 1xPBS containing 0.5 mM AEBSF.

The proteins bound to the beads were eluted in 40 μL of loading buffer at 85°C for 10 min, separated by SDS-PAGE, and transferred onto nitrocellulose membranes, which were incubated separately with anti-p50 or anti-p52, anti-RelA or anti-RelB, and anti-IκBα antibodies. After washing with 1x Tris-buffered saline with 0.1% Tween 20 (TBST) buffer three times for 5 min each, the nitrocellulose membranes were incubated with an appropriate secondary antibody for protein detection with a LI-COR near-infrared imaging system.

### Enzyme‐linked immunosorbent assay (ELISA)

IL-1β and IL-18 concentrations in THP1 cell supernatant were quantified by ELISA (Abcam, Cat. No. ab100562, ab46032) following the manufacturer’s instruction [20].

### Statistical analysis

Data are presented as mean±standard deviation (SD) unless otherwise stated. All experiments were performed in triplicate or as indicated in the figure legends. Differences among three or more groups were assessed by one-way factorial ANOVA with Newman-Keuls test (GraphPad Prism, La Jolla, CA). P values <0.05 were considered statistically significant.

## Results

### OxLDL increased NLRP3 inflammasome activity

**Fig 1:**
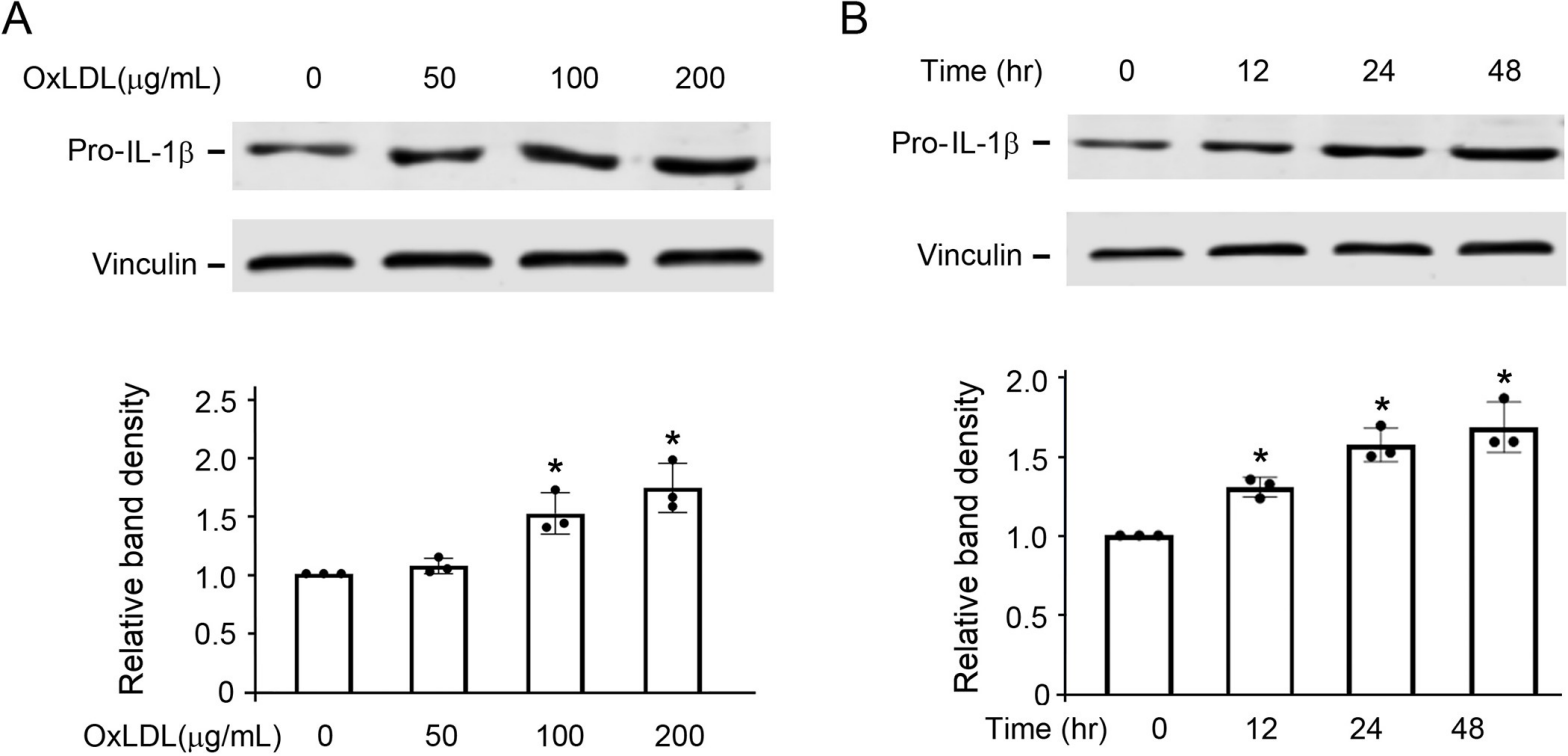
Exposure to oxLDL increases pro-IL-1β protein expression in a concentration- and time-dependent manner. (**A**) THP-1 cells were exposed to the indicated concentrations of oxLDL for 24 hr. (**B**) THP-1 cells were exposed to 100 μg/mL oxLDL at the indicated time points. The band densities of pro-IL-1β and vinculin bands (the latter as a loading control), were normalized with the bands in the vehicle-treated cells (0 μg/mL) from three independent experiments (n = 3/group), representing a total of seven replicates. *P<0.05 vs 0 hr, one-way ANOVA, Newman-Keuls test.

Based on observations from our and others’ previous work [20, 21], we chose oxLDL concentrations of 0, 50, 100, 200 μg/mL and duration of incubation of 0, 12, 24, and 48 hours to study the concentration-response and time course of oxLDL exposure on NLRP3 inflammasome activity in cultured THP-1 cells. As expected, oxLDL increased NLRP3 inflammasome activity, determined by the downstream protein expression of pro-IL-1β, in a concentration- (Fig 1A) and time-dependent (Fig 1B) manner. This increase was abrogated by pre-treatment with MCC950 (S1 Fig), a selective NLRP3 inhibitor [22].

### OxLDL increased PARP activity

**Fig 2:**
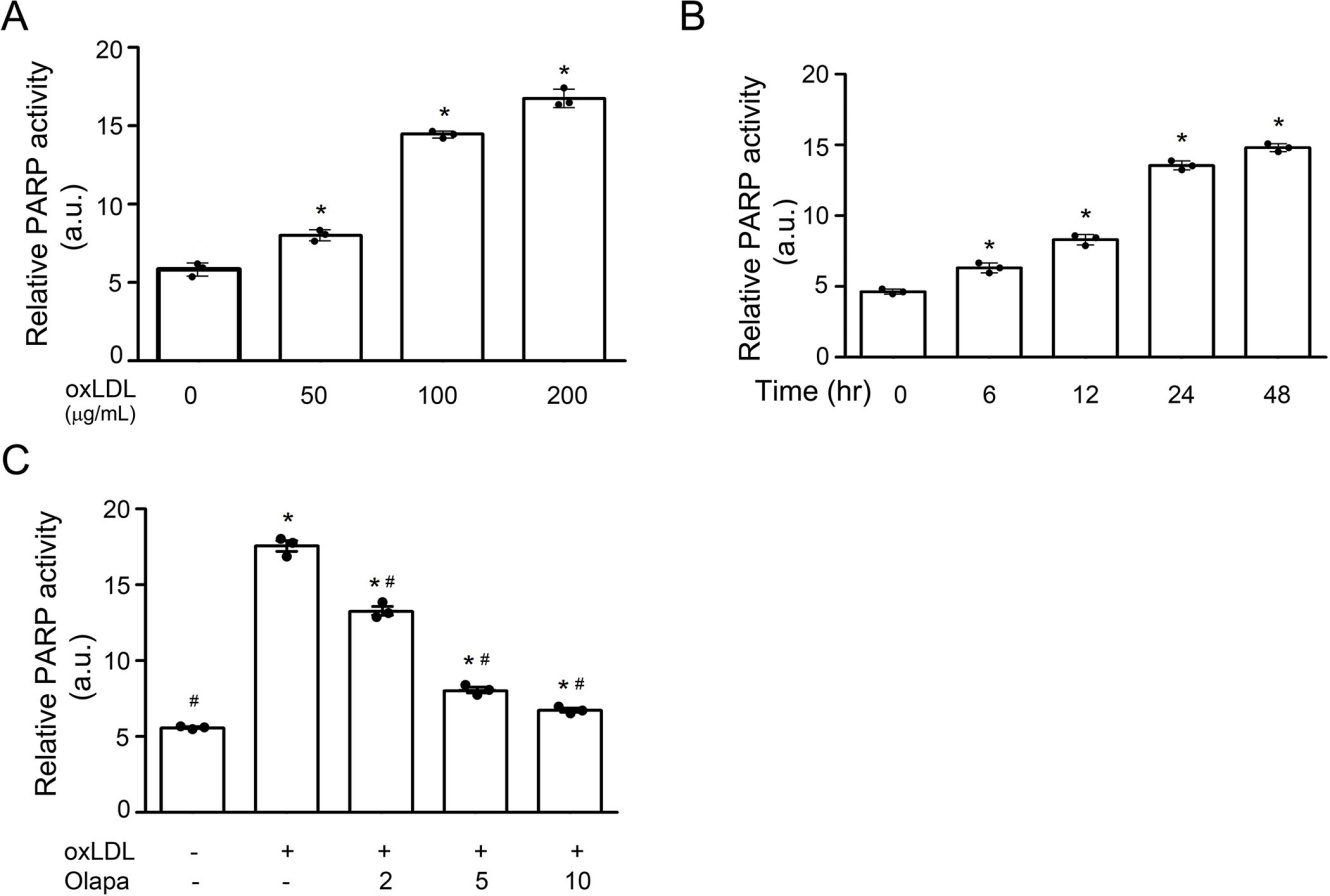
Olaparib inhibits oxLDL-mediated increase in PARP activity. (A) Human THP-1 monocytes were exposed to the indicated concentration of oxLDL for 24 hr. (B) THP-1 monocytes were exposed to 100 μg/mL oxLDL at the indicated time points. (C) THP-1 monocytes were exposed to oxLDL (100 μg/mL, 24 hr) without or with the PARP inhibitor olaparib (Olapa) at the indicated concentrations (μM, 24 hr). The data are from three independent experiments (n = 3/group). a.u., arbitrary unit. Median and SD values are shown. *P<0.05 vs vehicle (without oxLDL and Olapa), #P<0.05 vs oxLDL only, one-way ANOVA, Newman-Keuls test.

PARP is activated by oxidative stress [23]. Therefore, we investigated whether oxLDL increases PARP activity, determined by the amount of free ammonia generated upon cleavage of NAD^+^ during PARP-mediated poly-ADP-ribosylation [24]. Consistent with studies showing that PARP is activated by oxidative stress, oxLDL increased free ammonia in a concentration- (Fig 2A) and time-dependent (Fig 2B) manner.

Olaparib, a PARP inhibitor [16], inhibited the oxLDL-induced increase in PARP activity in a concentration-dependent manner (Fig 2C).

### PARP inhibition by olaparib attenuated the oxLDL-mediated increase in protein expression of NLRP3 inflammasomes

**Fig 3:**
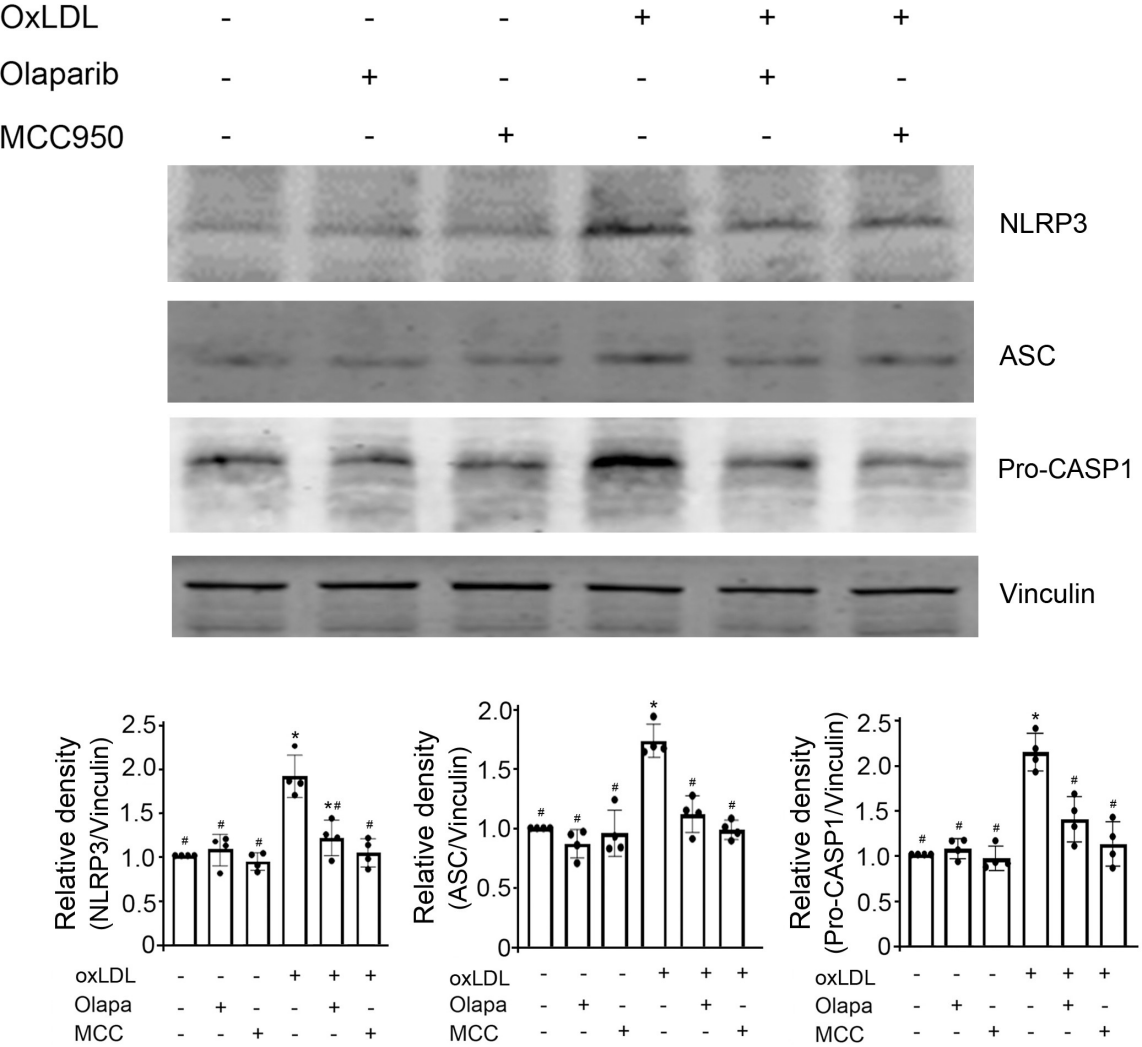
Olaparib inhibits oxLDL-mediated NLRP3 inflammasomes activity. THP-1 monocytic cells were exposed to oxLDL, the PARP inhibitor olaparib (Olapa), or the NLRP3 inhibitor MCC950 (MCC), individually or in combination as indicated. Proteins in cell lysates were detected with anti-NLRP3, anti-ASC, and anti-pro-caspase-1 (pro-CASP1) antibodies, as indicated. The band densities, relative to vinculin (loading control) were quantified using ImageJ (NIH, Bethesda, MD). N = 4/group, * P<0.05 vs vehicle (without oxLDL, Olapa, and MCC), ^#^P<0.05 vs oxLDL only, one-way ANOVA, Newman-Keuls test.

As expected, oxLDL enhanced the protein expression of NLRP3 inflammasome components, assessed by immunoblotting for NLRP3, ASC, and caspase-1 (Fig 3). By contrast, olaparib attenuated the oxLDL-induced increase in protein expression of NLRP3, ASC, and caspase-1 (Fig 3). MCC950, an NLRP3 inhibitor that served as a positive control, also attenuated the oxLDL-mediated increase in the protein expression of NLRP3 inflammasomes. Of note, neither MCC950 nor olaparib, by themselves, had an effect on the protein expression of NLRP3 inflammasomes (Fig 3). These data suggested PARP inhibition by olaparib attenuated the oxLDL-mediated increase in NLRP3 inflammasomes activity.

### PARP inhibition by olaparib impaired the oxLDL-mediated increase in mitochondrial reactive oxygen species

**Fig 4:**
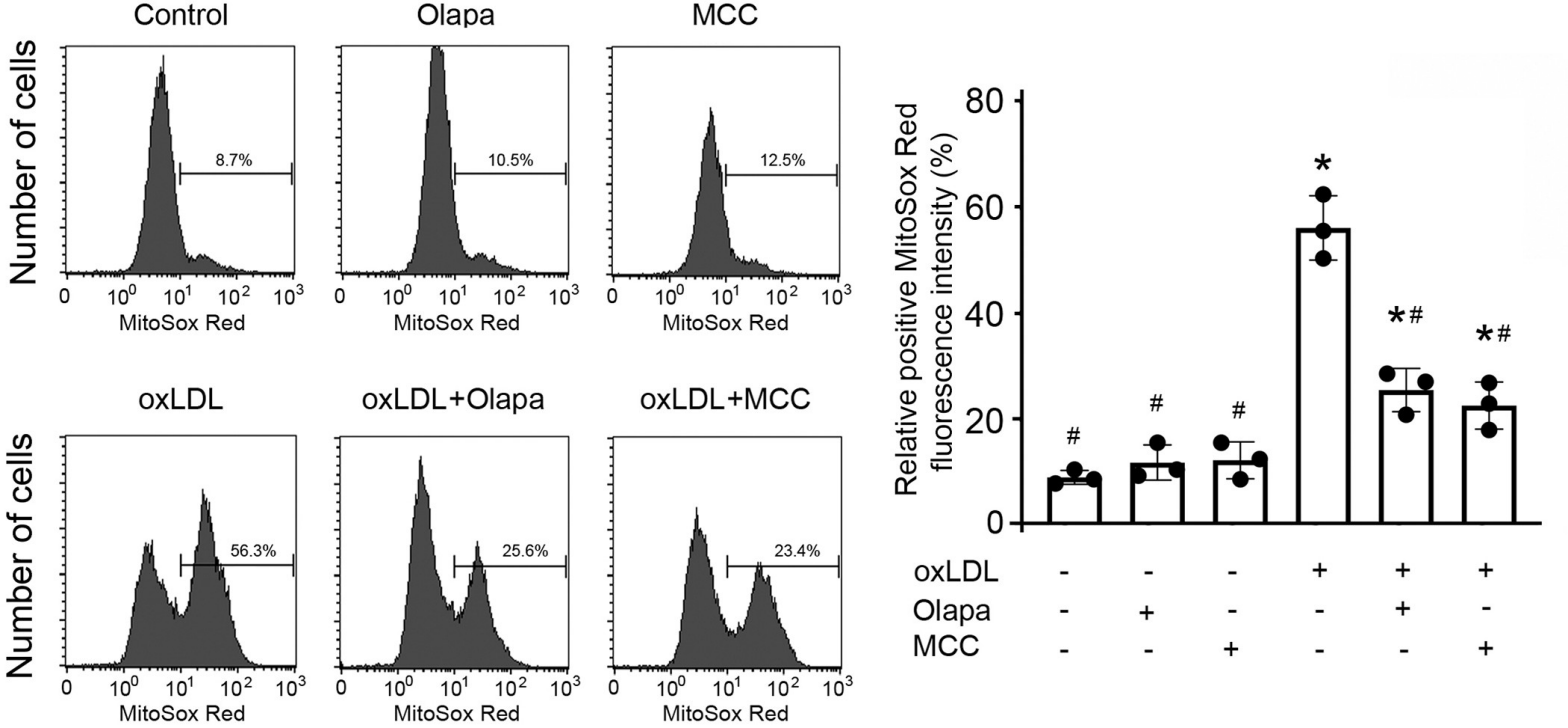
Olaparib inhibits oxLDL-enhanced mitochondrial ROS production. THP-1 monocytes were exposed to oxLDL, the PARP inhibitor olaparib (Olapa), or the NLRP3 inhibitor MCC950 (MCC), individually or in combination, as indicated. Mitochondrial ROS production was measured by MitoSox Red fluorescence intensity using flow cytometry. Median and SD values are summarized from three independent experiments (n = 3/group). *P<0.05 vs vehicle, #P<0.05 vs oxLDL only, one-way ANOVA, Newman-Keuls test.

Mitochondrial reactive oxygen species (mito-ROS) activate NLRP3 inflammasomes [25]. As expected, oxLDL exposure increased mitochondrial ROS production in THP-1 monocytes, detected by MitoSox Red, using FACS flow cytometry (Fig 4). By contrast, olaparib significantly reduced the oxLDL-mediated increase in mito-ROS production (Fig 4), which was corroborated by its inhibitory effect on the protein expression of NLRP3 inflammasomes (Fig 3). Consistent with this finding, MCC950 also reduced the oxLDL-mediated increase in mito-ROS production (Fig 4).

### PARP inhibition by olaparib attenuated the oxLDL-mediated increase in IL-1β and IL-18 protein expression

**Fig 5:**
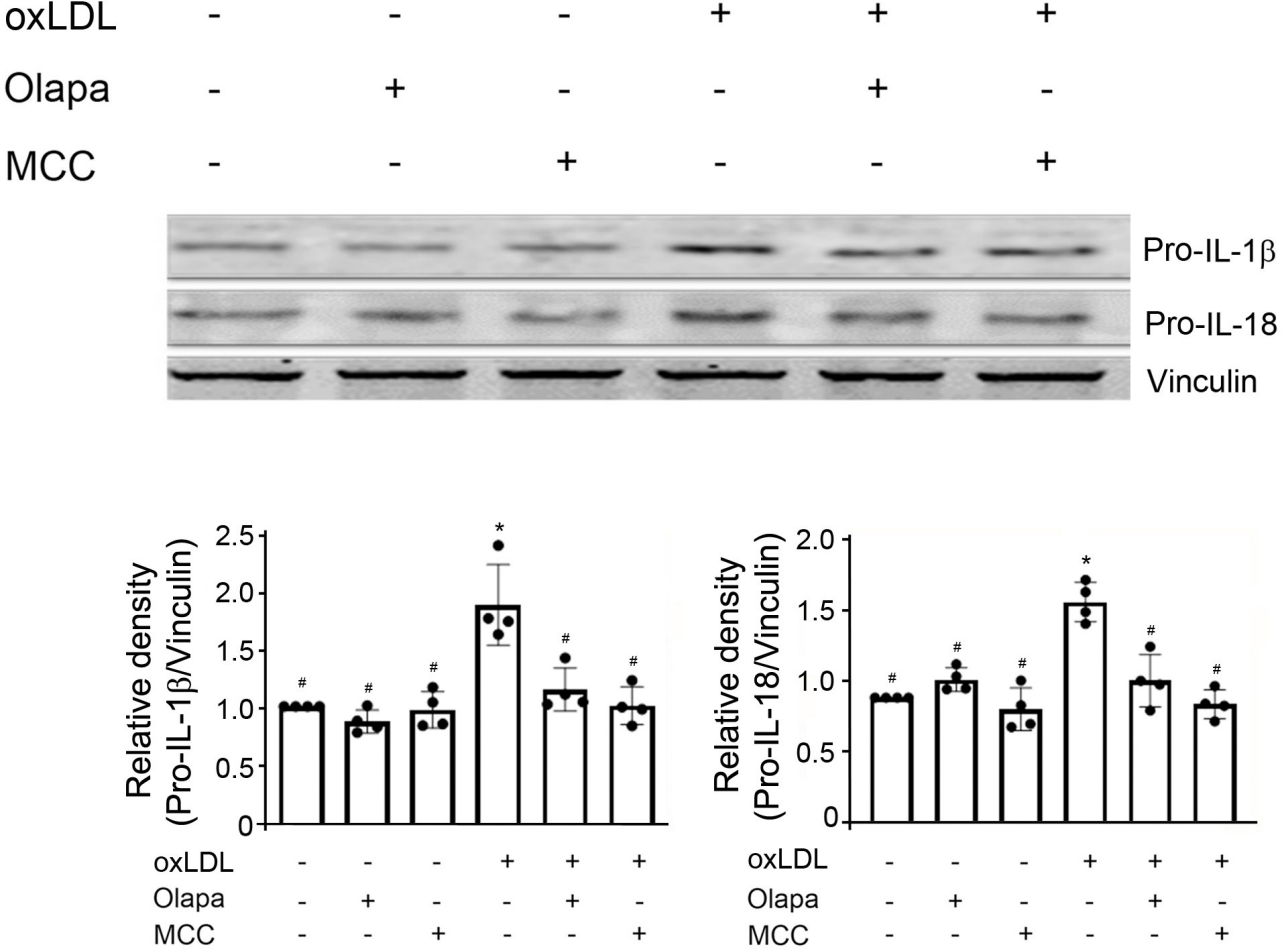
Olaparib attenuates the oxLDL-mediated increase in pro-IL-1β and pro-IL-18 protein expression. THP-1 monocytes were exposed to oxLDL, the PARP inhibitor olaparib (Olapa), or the NLRP3 inhibitor MCC950 (MCC), individually or in combination as indicated. Cell lysates were subjected to SDS-PAGE and immunoblotted with anti-pro-IL-1β (top), anti-pro-IL-18 (middle) or anti-vinculin antibody (bottom, for loading control). The densities of pro-IL-1β, pro-IL-18 over vinculin were quantified by densitometry and normalized to vehicle. The band densities, relative to vinculin, were quantified using ImageJ (NIH, Bethesda, MD). N = 4/group, *P<0.05 vs vehicle, #P<0.05 vs oxLDL only, one-way ANOVA, Newman-Keuls test.

Following activation of NLRP3 inflammasomes, caspase-1 is activated and catalyzes the maturation of IL-1β and IL-18 from pro-IL-1β and pro-IL-18, respectively [26]; this is followed by secretion of mature IL-1β and IL-18 [26]. As expected, oxLDL increased the protein expressions of pro-IL-1β and pro-IL-18 (Fig 5) and the release of mature IL-1β and IL-18 into the supernatant (S2 Fig). The PARP inhibitor, olaparib, decreased the oxLDL-mediated increase in protein expression of pro-IL-1β and pro-IL-18 (Fig 5) and mature IL-1β and IL-18 into the supernatant (S2 Fig). Olaparib, in the absence of oxLDL, had no effect on the protein expression of pro-IL-1β and pro-IL-18 (Fig 5) and mature IL-1β and IL-18 into the supernatant (S2 Fig). The effects of the NLRP3 inhibitor MCC950 were similar to the effects of the PARP inhibitor olaparib (Fig 5) (S2 Fig).

### PARP inhibition by olaparib attenuated oxLDL-induced monocyte adhesion and foam cell formation that are important in the atherosclerotic process

**Fig 6:**
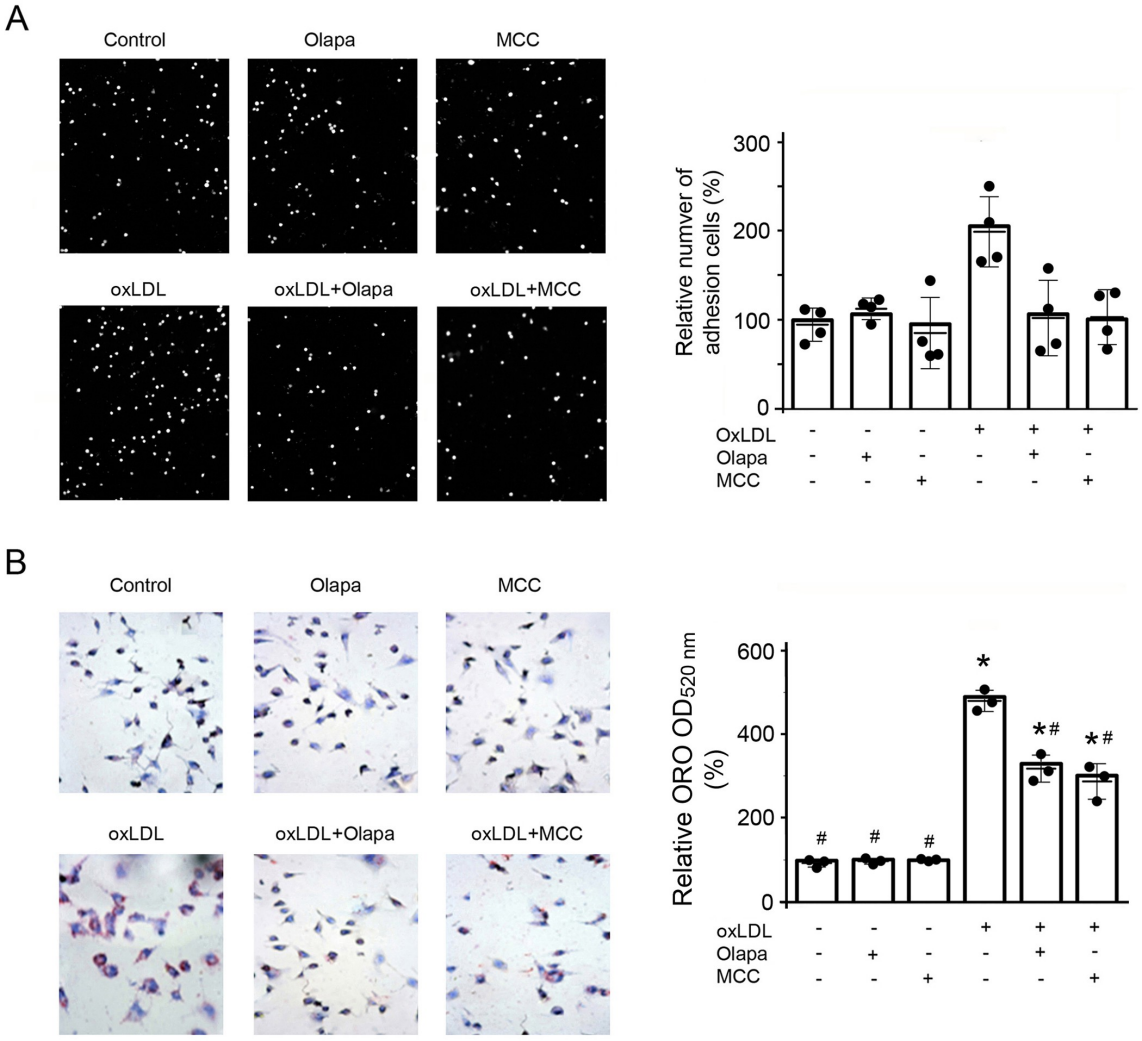
Olaparib inhibits oxLDL-induced monocyte adhesion and foam cell formation. (A) THP-1 monocytes were exposed to oxLDL, the PARP inhibitor olaparib (Olapa), or the NLRP3 inhibitor MCC950 (MCC) as indicated. The monocytes attached to human umbilical vein endothelial cells (HUVECs) were counted using an inverted light microscope and analyzed by ImageJ software (NIH, Bethesda). Data are from four independent experiments (n = 4/group). *P<0.05 vs vehicle, #P<0.05 vs oxLDL only, one-way ANOVA, Newman-Keuls test. (B) THP-1 cells were exposed to PMA followed by compounds as in (A) as described in Methods section. Formation of foam cells was assayed by oil red-O staining and imaged by an inverted light microscope. ORO, oil red-O staining. Data are from three independent experiments (n = 3/group). Median and SD values are shown. *P<0.05 vs vehicle, #P<0.05 vs oxLDL only, one-way ANOVA, Newman-Keuls test.

The adhesion of monocytes to the vascular endothelium is important in the initiation and progression of atherosclerosis [27]. OxLDL markedly increased the adhesion of monocytes to HUVEC monolayers and this was decreased by either olaparib or MCC950 (Fig 6A). Neither olaparib nor MCC950, by itself, had an effect on the adhesion of monocytes to the HUVECs under basal conditions (Fig 6A).

Activated NLRP3 inflammasomes within macrophages participate in the pathogenesis of atherosclerosis by increasing formation of foam cells [28]. We, therefore, investigated the consequences of PARP inhibition on foam cell formation. As expected, oxLDL markedly increased foam cell formation, identified by ORO, which was decreased by either the PARP inhibitor olaparib or MCC950 (Fig 6B). Neither olaparib nor MCC950, by itself, had an effect on foam cell formation under the baseline condition (Fig 6B).

### PARP inhibition by olaparib inhibited oxLDL-mediated NF-κB activity through the canonical and non-canonical NF-κB pathway

**Fig 7:**
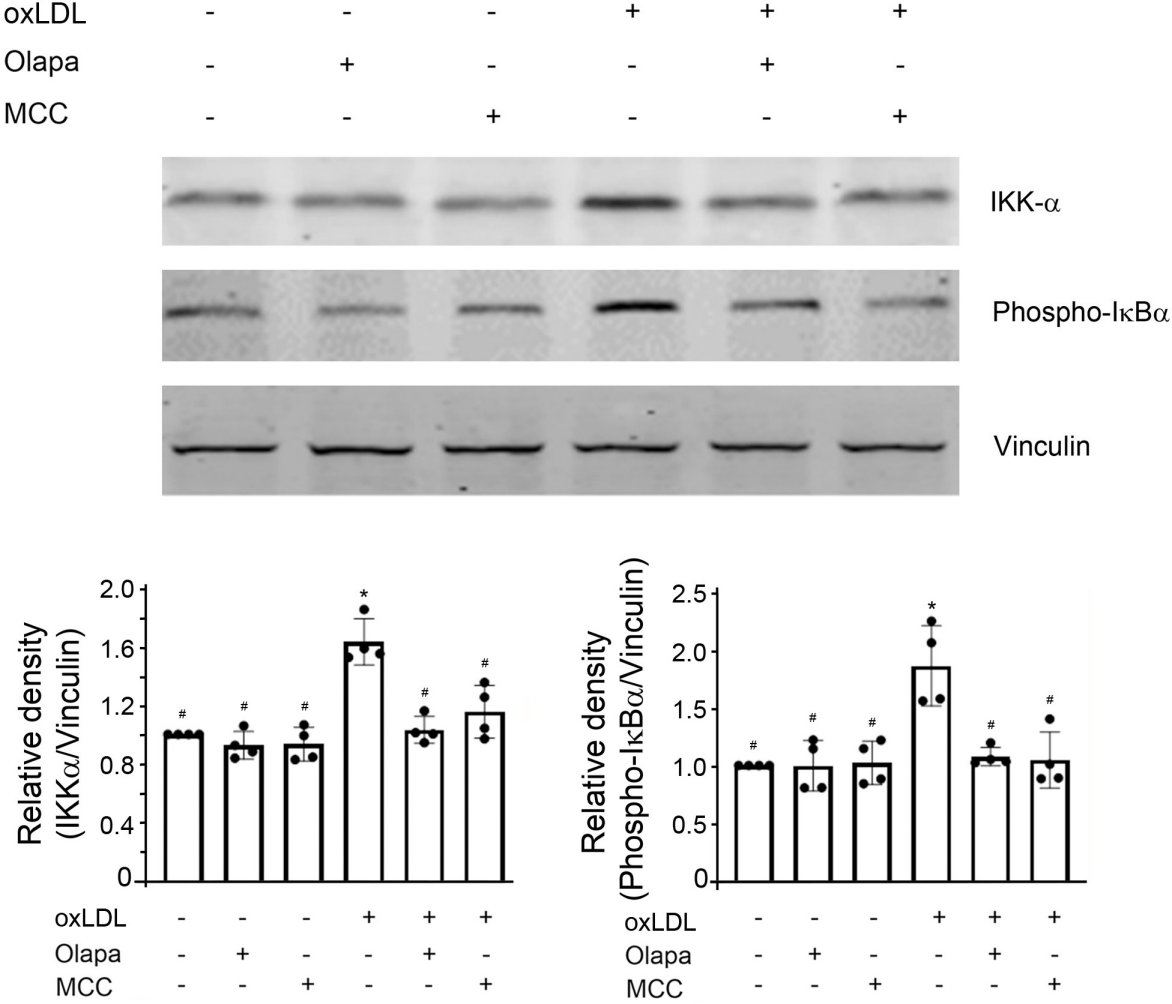
Olaparib inhibits oxLDL-induced increase in NF-κB activity. The THP-1 monocytes were exposed to oxLDL, the PARP inhibitor olaparib (Olapa), or the NLRP3 inhibitor MCC950 (MCC) as indicated. The cell lysates were subjected to SDS-PAGE and immunoblotted with anti-IKK-α (top), anti-phospho-IκBα (middle) or anti-vinculin antibody (bottom, for loading control), as indicated. The band densities, relative to vinculin, were quantified using ImageJ (NIH, Bethesda, MD). N = 4/group, *P<0.05 vs vehicle, #P<0.05 vs oxLDL only, one-way ANOVA, Newman-Keuls test.

Activation of NLRP3 inflammasomes requires two stimuli: a priming stimulus and an activating stimulus. NF-κB activation is important in priming NLRP3 inflammasomes [11, 29]. The critical step in NF-κB activation is the phosphorylation of IκBα by IKK complex [13, 14, 30]. Therefore, we next investigated the effect of olaparib on protein expression of IKK and phospho-IκBα. The expression of IKK-α and phospho-IκBα were markedly increased by oxLDL, an effect that were decreased by either olaparib or the inflammasome inhibitor MCC950 (Fig 7). Neither olaparib nor MCC950, by itself, had an effect on the IKK-α protein expression or IκBα phosphorylation (Fig 7). These results indicate that Olaparib inhibited oxLDL-mediated increase in NF-κB activity.

**Fig 8:**
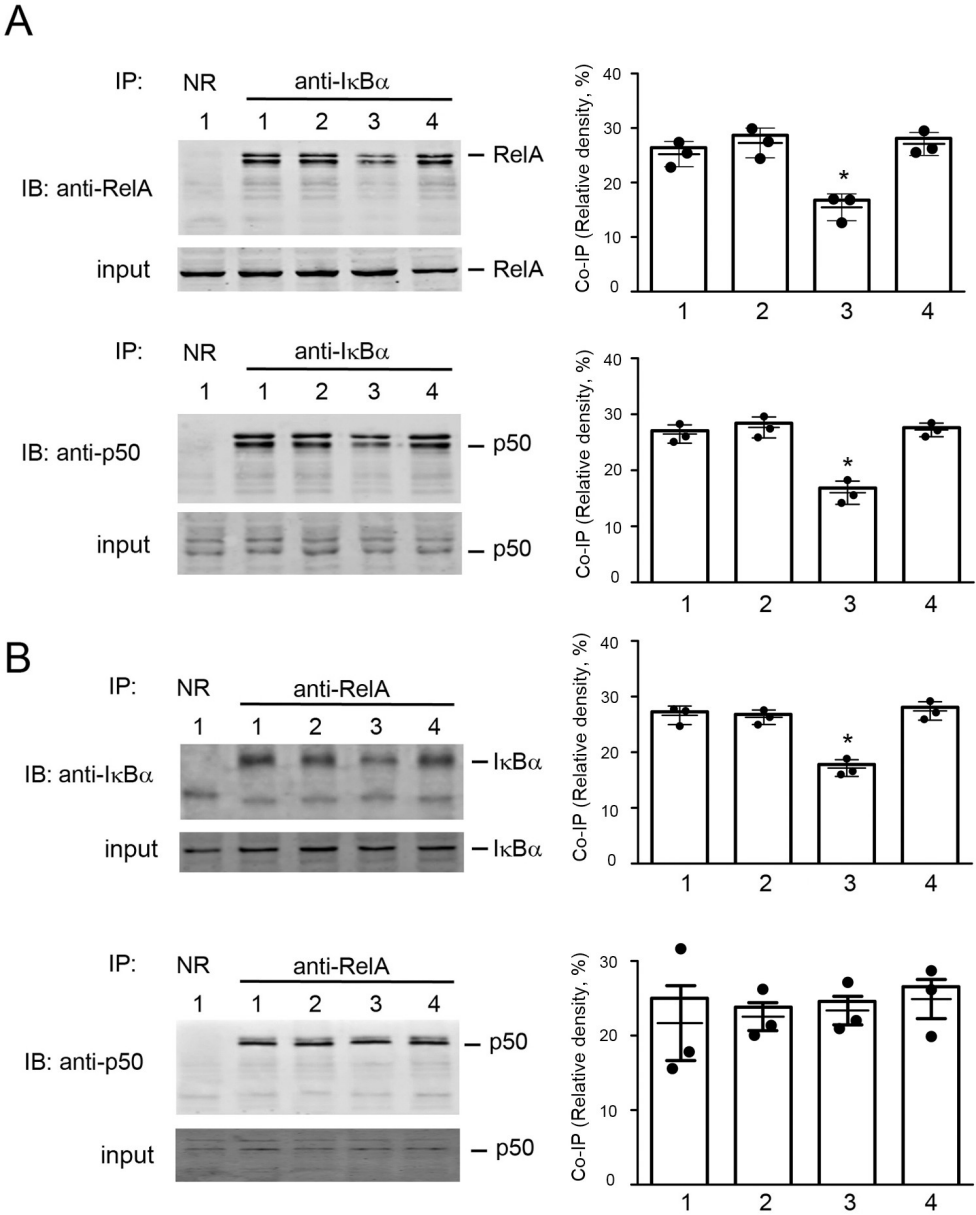
Olaparib reverses the oxLDL-mediated decrease in co-immunoprecipitation of IκBα with RelA or p50. Cell lysates were obtained from THP-1 monocytes exposed to reagents as indicated: Lane 1, vehicle; Lane 2, PARP inhibitor olaparib (5 mM, 24 hr); Lane 3, oxLDL (100 mg/mL, 24 hr) or Lane 4, combination of oxLDL and olaparib. Protein complexes, co-immunoprecipitated with the indicated antibodies, were separated by SDS-PAGE and immunoblotted with the antibodies, as indicated. NR, normal rabbit IgG. Relative band densities from three independent experiments were analyzed. (A) OxLDL decreased co-immunoprecipitation of IκBα with both RelA and p50, and olaparib reversed the effect of oxLDL on the dissociation of IκBα with RelA and p50. (B) OxLDL decreased co-immunoprecipitation of RelA with IκBα but not with p50, and olaparib reversed the effect of oxLDL on the co-immunoprecipitation of RelA with IκBα. The data are from three independent experiments (n = 3/group). *P<0.05 vs vehicle (Lane 1), one-way ANOVA, Newman-Keuls test.

NF-κB can be activated through canonical or non-canonical pathway [14]. The RelA/p50 heterodimer is released from binding with IκBα through canonical pathway upon phosphorylation of IκBα [14]. Consistently, anti-IκBα co-immunoprecipitated with RelA and p50 (Fig 8A), and anti-RelA complementarily co-immunoprecipitated IκBα (Fig 8B) and p50 (Fig 8B). The co-immunoprecipitations of IκBα with RelA or p50 were significantly decreased with oxLDL exposure, but these were restored by the combined exposure to oxLDL and olaparib (Fig 8).

These results indicate that oxLDL activated NF-κB, leading to the dissociation of IκBα from the RelA/p50 heterodimer. By contrast, olaparib inhibited NF-κB activation and the dissociation of IκBα from RelA/p50; IκBα then reassembled with the RelA/p50 heterodimer. Interestingly, the co-immunoprecipitation of RelA with p50 was not affected by oxLDL and/or olaparib (Fig 8B), indicating that the stability of RelA/p50 heterodimers was not affected by oxLDL exposure or PARP inhibition. These co-immunoprecipitation results suggest that oxLDL and PARP inhibition regulates NF-κB activity through modulating the disassembly and reassembly of NF-κB subunits and the nuclear transport of RelA/p50 subunit.

**Fig 9:**
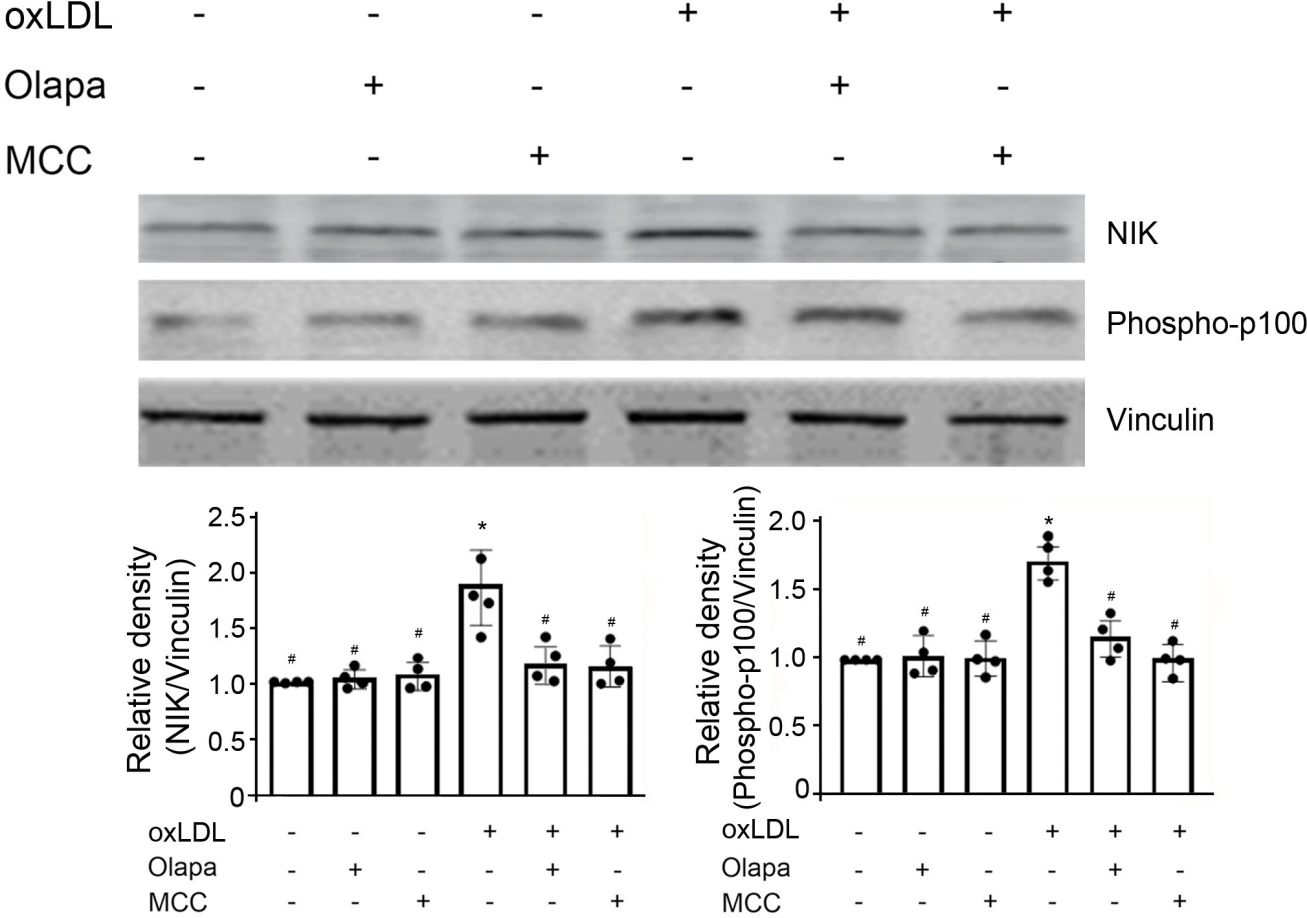
Olaparib inhibits oxLDL-induced NIK protein expression and p100 phosphorylation. The THP-1 monocytes were exposed to oxLDL, the PARP inhibitor olaparib (Olapa), or the NLRP3 inhibitor MCC950 (MCC), as indicated. The cell lysates were subjected to SDS-PAGE and immunoblotted with anti-NIK (top), anti-phospho-p100 (middle) or anti-vinculin antibody (bottom, for loading control), as indicated. The band densities, relative to vinculin, were quantified using ImageJ (NIH, Bethesda, MD). N = 4/group, *P<0.05 vs vehicle, #P<0.05 vs oxLDL only, one-way ANOVA, Newman-Keuls test.

In addition to the canonical pathway, NF-κB activation also occurs via a non-canonical NF-κB signaling pathway [31]. Therefore, we investigated the effects of oxLDL and olaparib on non-canonical NF-κB activation. The central signaling component of the non-canonical NF-κB pathway is that of NF-κB-inducing kinase (NIK), which induces p100 phosphorylation via the activation of the kinase IKKα. Ox-LDL increased NIK protein expression and the phosphorylation of p100 (Fig 9). Either olaparib or MCC950 attenuated the ox-LDL-mediated increase in NIK and phospho-p100 protein expression (Fig 9), whereas both olaparib and MCC950, by themselves, had no effect on NIK and phospho-p100 protein expressions (Fig 9). These results indicate that olaparib inhibits ox-LDL-mediated increase in NF-κB activation also through the non-canonical NF-κB pathway.

Anti-IκBα co-immunoprecipitated with RelB and p52 (S3 Fig), and anti-RelB complementarily co-immunoprecipitated with IκBα and p52 (S3 Fig). The co-immunoprecipitations of IκBα with RelB and p52 were decreased with oxLDL exposure, and these were restored by the combined exposure of oxLDL and olaparib (S3 Fig). These results indicate that PARP inhibition by olaparib inhibited oxLDL-mediated activation of NF-κB through the non-canonical pathway, leading to the dissociation of IκBα from the RelB/p52 heterodimer.

### PARP inhibition by olaparib suppressed NF-κB-targeted gene *VCAM1* coded protein expression

**Fig 10:**
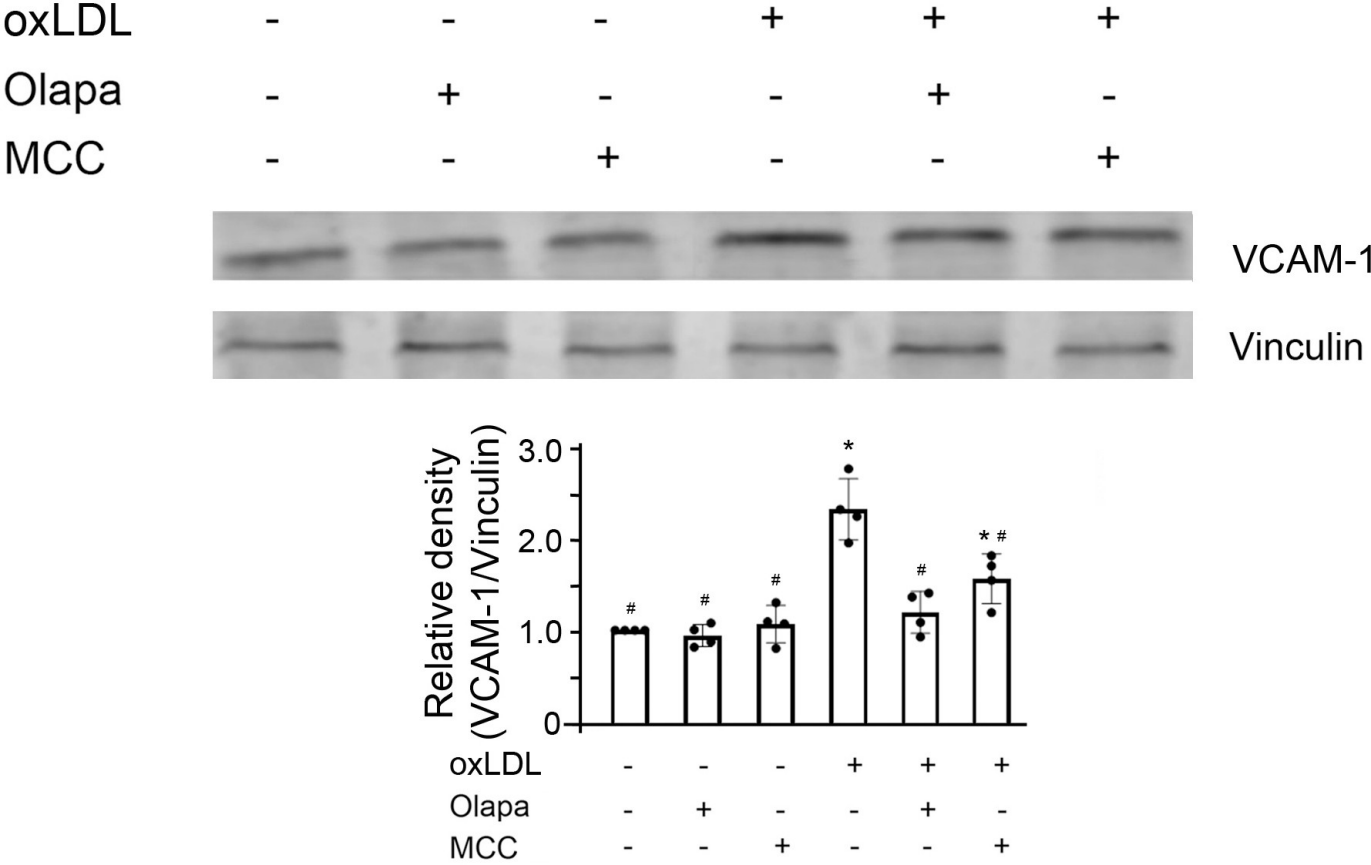
Olaparib suppresses oxLDL-enhanced NF-κB target-gene VCAM-1 protein expression in THP-1 monocytes. The cells were exposed to oxLDL, the PARP inhibitor olaparib (Olapa), or the NLRP3 inhibitor MCC950 (MCC), as indicated. The cell lysates were subjected to SDS-PAGE and immunoblotted with anti-VCAM-1 (upper panel) or anti-vinculin antibody (lower panel). The band densities, relative to vinculin (loading control), were quantified using ImageJ (NIH, Bethesda, MD). N = 4/group, * P<0.05 vs vehicle, #P<0.05 vs oxLDL only, one-way ANOVA, Newman-Keuls test. VCAM-1, vascular cell adhesion molecule 1.

*VCAM-1*, encoding vascular cell adhesion molecule-1, is a well-established NF-κB target gene and the protein product is associated with atherosclerosis [32]. We investigated whether olaparib affected the protein expression of VCAM-1. OxLDL increased VCAM-1 protein expression which was suppressed by either olaparib or MCC950 (Fig 10), consistent with their roles in the pathogenesis of atherosclerosis (Fig 6). Neither olaparib nor MCC950, alone, had effect on the protein expression of VCAM-1.

## Discussion

This study demonstrated that oxLDL, acting on THP1 monocytes, increased NLRP3 inflammasome and PARP activity, and that PARP inhibition by olaparib inhibited oxLDL-induced PARP and NLRP3 inflammasome activity. The effect of olaparib was in direction and magnitude similar to that caused by MCC950, a potent NLRP3 inhibitor [22]. Additional experiments showed that PARP inhibition by olaparib inhibited the production of Mito-ROS, which is upstream of NLRP3 inflammasome activation, and inhibited subsequent downstream IL-1β and IL-18 protein expressions. This led to the inhibition of monocyte adhesion to endothelial cells and foam cell formation.

NF-κB activation occurs via both canonical and non-canonical NF-κB signaling pathways. The canonical pathway mediates the activation of p50, RelA, and c-REL through activation of IκB kinase (IKK) complex (comprised of heterodimer of IKKα and IKKβ catalytic subunits (and regulatory subunit, IKKγ/NEMO) [33], whereas the non-canonical NF-κB pathway activates p100-sequestered NF-κB members, predominantly p52 and RelB [31]. NIK is the central component of non-canonical pathway, which induces phosphorylation of p100 through IKKα activation [31]. The canonical and non-canonical NF-κB pathways are interlinked [34]. Phosphorylation of IKKα and IKKβ occurs concurrently in endogenous systems, and phosphorylated IKKβ subunit phosphorylates its adjacent IKKα subunit [33, 34]. The olaparib-mediated decrease in protein expression of NLRP3 inflammasomes is associated with decreased NF-κB activity. PARP inhibition by olaparib attenuated the oxLDL-mediated increase in IκBα phosphorylation, which in turn restored the sequestration of IκBα with NF-κB RelA/p50 and RelB/p52 heterodimers. The resulting decrease in the expression of the NF-κB target gene *VCAM-1* is expected to curtail the initiation and progression of atherosclerosis.

Atherosclerosis is a chronic inflammatory disease, characterized by local and systemic inflammation involving the innate immune system, with monocytes/macrophages playing an important role [35]. In early-stage atherosclerosis, monocytes/macrophages recognize and internalize oxLDL [7]; NLRP3 inflammasomes are activated and macrophages differentiate into foam cells. The inhibition of NLRP3 inflammasomes by MCC950 or by silencing NLRP3 with a specific siRNA [20] reduces the production of pro-inflammatory cytokines and prevents the transformation of macrophages into foam cells, by suppression of oxLDL uptake and enhancement of cholesterol efflux. In late-stage atherosclerosis, NLRP3 inflammasomes induce macrophage cell death by pyroptosis, releasing IL-1β, IL-18, and inflammatory lipids, which increase plaque size and vulnerability to vessel rupture [8, 36].

ROS play a crucial role in the activation of NLRP3 inflammasomes [37]. A recent study has demonstrated that ATP-induced ROS production increases NLRP3 inflammasome activity through interaction with thioredoxin-interacting protein [38]. There are two major intracellular ROS sources: mitochondria-derived ROS and NADPH oxidase-derived ROS [39]. Experiments in multiple cell systems show that activation of NLRP3 inflammasomes is independent of NADPH oxidase [40, 41]. Therefore, mitochondria-derived ROS are critical for NLRP3 inflammasome activation [42, 43]. The present study measured mito-ROS using MitoSox Red with flow cytometry [18], demonstrating that oxLDL increased mito-ROS production and activated NLRP3 inflammasomes, and that olaparib attenuated oxLDL-mediated increase in mito-ROS and activation of NLRP3 inflammasomes. This indicates that the inhibition of PARP by olaparib on oxLDL-mediated NLRP3 inflammasome activity is via the canonical pathway and is mito-ROS dependent. Although mito-ROS are widely detected with MitoSox Red probe at low concentrations, it is recommended to independently verify mito-ROS production [44]. It is a limitation of this study that only a single method was used to determine mito-ROS level without normalization with factors like plasma membrane potential and mitochondrial membrane potential that affect the fluorescence probe accumulation [44]. Liquid chromatography-tandem mass spectrometry, which overcomes limitations of spectral overlap and nonspecific fluorescence spectra [45], will be used in the future studies.

In addition to oxidative stress associated with mito-ROS, mitochondrial dysfunction (*e*.*g*, mtDNA release, defective mitochondrial membrane potential, aberrant mitochondrial dynamics, impaired mitochondrial homeostasis) are also major drivers for the activation of NLRP3 inflammasomes, intensifying the NLRP3 inflammasome-mediated pro-inflammatory responses [25, 41, 46]. Whether or not olaparib regulates NLRP3 inflammasome activity through mitochondrial dynamics, membrane integrity and homeostasis, and noncanonical pathway such as caspase-11 warrants further investigation.

Oxidative stress activates PARP [23]. The present study showed that oxLDL increased PARP activity in a concentration- and time-dependent manner. ROS overproduction damages nucleobases and sugar moieties in DNA, forming various oxidized bases and transition and transversion mutations, and DNA breaks [47], all of which pose a serious threat to genome integrity [48]. PARP enzymes sense these forms of DNA damage, so that proteins containing PAR-binding modules can be recruited to sites of DNA breaks in a PAR-dependent manner [48]. PARP-1 catalyzes the poly ADP-ribosylation reaction by transferring ADP-ribose to target proteins, resulting in long, branched chains of poly ADP-ribose [49]. These negatively-charged polymers, acting through NF-κB [13, 30], induce diverse pro-inflammatory responses in monocytes/macrophages and other immune cells [50, 51].

NF-κB is the master inducer of proinflammatory responses linked to oxidative stress [14, 29]. Inhibition of NF-κB activation, whether accomplished pharmacologically or by gene knockout of IκB or IKK, decreases the expression of pro-inflammatory cytokines, chemokines, intercellular adhesion molecule 1, and other inflammation-related molecules [13, 29, 30]. Accumulating evidence shows that PARP positively regulates NF-κB activity and increases inflammatory responses [52]. The mechanisms by which PARP increases NF-κB activity are not well understood. It has been reported that PARP-1 transiently interacts with NF-κB and acts as a coactivator of NF-κB in regulating gene expression [53, 54]. PARP-1 facilitates NF-κB binding to specific DNA elements located in promoters and enhancers of target genes [55]. PARP-1 also regulates the activities of other transcription factors, increasing NF-κB-dependent expression of target genes [54]. Binding of PARP-1 with p300/CBP allows the acetylation of PARP-1 and facilitates binding of p300 and p50 subunit to p300/CBP, resulting in the full activation of NF-κB and thereby promoting the transcription of inflammatory mediators [3]. PARP1-mediated PARylation of histones at transcriptionally active chromatin regions and targeted gene promoters facilitates NF-κB recruitment to these promoters [56]. It is established that the DNA-binding of PARP, rather than the catalytic activity of PARP on PARylation of NF-κB, enhances the transcriptional activity of PARP [54, 57]. Consistent with this model is the finding in the current study that PARP1 is crucial for the assembly of NLRP3 inflammasomes and their activation.

The deactivation of PARP results in inhibition of NF-κB and decrease in proinflammatory cytokine expression [52, 53]. The present results showed that oxidative stress induced by oxLDL increased mito-ROS production, PARP activity, and the phosphorylation of IκBα. These factors caused the dissociation of IκBα from RelA/p50 or RelB/p52 and the activation of NF-κB. PARP inhibition by olaparib attenuated oxLDL-induced IκBα phosphorylation. These actions restored the association of IκBα with RelA/p50 and RelB/p52 heterodimers and consequently prevented NF-*κ*B nuclear translocation and activation.

The present study identified an additional and novel mechanism for PARP-mediated NF-*κ*B activation, through its modulation of its subunit IκBα with RelA/p50 and RelB/p52 assembly. PARP-mediated NF-*κ*B activation occurs in the cytoplasm and for the first time relates this effect to the pathogenesis of atherosclerosis. This mechanism operates upstream of NF-*κ*B nuclear translocation. PARP-1 is responsible for 80–90% of total PARP activity [2, 4] and was initially considered to be located exclusively in the nucleus [4, 42]. However, recent studies suggest that PARP-1 is also present and active in the cytosol [58]. Among the 17 human PARPs [59], PARP-1, -2, -5A, -5B, and -6 catalyze poly-ADP-ribosylation [50] and may participate in the oxLDL-mediated NF-*κ*B activation. Olaparib forms a hydrogen bond with PARP-1 residue Y896. Olaparib also has a hydrophobic interaction with H862 of the PARP-1 catalytic HYE amino acid triad (histidine-tyrosine-glutamic acid) HYE forms both hydrophobic interactions and hydrogen bonds with other residues in the binding pocket of PARP1 [60]. Five PARPs (PARP-1, -2, -4, -5A, -5B) share the conserved HYE tripeptide [3]. Therefore, olaparib may bind the cytosolic domain of these PARPs, inhibit PARP activity, and abrogate the oxLDL-induced NF-*κ*B nuclear translocation and activation.

Recent evidence indicates that NF-*κ*B activation is promoted, not only by poly ADP-ribosylated PARPs, but also by certain mono-ADP-ribosylated PARPs (*e*.*g*., PARP-10, -12) [61, 62]. In mouse bone marrow-derived macrophages, NLRP3 is directly poly-ADP-ribosylated by PARP-1 [38], which provides an additional mechanism for NLRP3 inflammasome activation by PARP-1. Nevertheless, identification of potential PARP-1 substrates and the PARP isoform(s) responsible for the oxLDL-mediated NF-*κ*B translocation and activation in THP-1 cells warrant further investigation.

In addition to olaparib and other exogenous PARP inhibitors, endogenous enzymes, such as poly-(ADP-ribose) glycohydrolase (PARG), ADP-ribosyl hydrolase-3 (ARH3), and macrodomain-containing terminal ARHs, including MacroD1, MacroD2, and C6orf130 enzymes, suppress PARP activity [2, 49]. Under oxidative stress, ribosylated cytoplasmic polymers, the product of long, branched chain of poly-ADP ribose from PARP activity, are translocated from the nucleus to the cytoplasm. There, poly-ADP ribose promotes release of apoptosis-inducing factors from the outer mitochondrial surface into the cytoplasm. Poly-ADP ribose is also translocated back to the nucleus and induces DNA fragmentation and chromatin condensation, resulting in cellular necrosis [2, 49]. Furthermore, PARP hyperactivation consumes NAD^+^ and depletes cellular ATP, also promoting cell death [3, 4].

OxLDL induces cleavage of PARP-1 protein in a concentration- and time-dependent manner in THP-1 cells, which indicates potential roles of PARP-mediated parthanatos (a form of programed cell death) in the death of monocytes/macrophages and foam cells, lipid release, and subsequent development of atherosclerosis. Macrophage phenotypes are heterogeneous, with extremes of classically activated (M1) and alternatively-activated (M2) macrophages; M1 is pro-inflammatory and M2 is anti-inflammatory [63]. Particular stages of atherosclerosis progression are associated with the presence of particular macrophage subtypes. Thus, M1 macrophages dominate in advanced atherosclerosis and M2 macrophages accumulate in plaque regression [64]. Induction of specific macrophage functions is probably closely related to the activities of PARP- and PAR-degrading enzymes in the local environment that orchestrate macrophage function.

Conventionally, PARP is known for its roles in DNA repair, stress response, cell division and differentiation, and cancer development and progression. The present study has demonstrated the role of PARP in modulating inflammatory responses through assembly/disassembly of NF-κB subunits (IκBα with either RelA/p50 or RelB/p52) in monocytes/macrophages, major participants in the pathogenesis of atherosclerosis. The phagocytosis of oxLDL activates NLRP3 inflammasomes in THP-1 cells; here we show that PARP has a similar effect. PARP inhibition by olaparib, a drug approved by the US Food and Drug Administration for *BRCA*-mutated ovarian cancer. Olaparib and other PARP inhibitors have been widely studied in preclinical and clinical applications in oncologic and non-oncologic diseases.

In C57BL/6 mice subjected to cecal ligation and puncture, olaparib attenuates circulating IL-1β levels, exerts anti-inflammatory effects, and plays organ protective roles, without adversely affecting DNA integrity [65]. In BALB/c mice, intraperitoneal injection of olaparib ameliorates ovalbumin-induced oxidative stress and NF-κB activation and attenuates maturation of IL-1β and NLRP3 inflammasome activity [66]. In transgenic R6/2 mice, intraperitoneal administration of olaparib reduces NLRP3 protein expression in striatal neurons, and olaparib prevents the loss of parvalbuminergic and calretininergic neurons [67]. In this study, olaparib attenuated the oxLDL-mediated increase in NLRP3 inflammasome activity, and also inhibited subsequent adhesion of monocytes to endothelial cells and inhibited macrophage foam cell formation. Whether or not olaparib has a role in attenuation of NLRP3 inflammasome activity in *Apoe*^-/-^ or *Ldlr*^-/-^ mouse, pig or other atherosclerosis animal models warrants further investigation.

## Conclusion

The data presented here demonstrate that PARP inhibition inhibits dissociation of the IκBα subunit from RelA/p50 and RelB/p52 heterodimers in monocyte/macrophages. PARP inhibition also inhibits NLRP3 inflammasome activity induced by NF-κB and oxidative stress in monocytes/macrophages. These findings suggest that PARP inhibition combined with immunomodulators could have a therapeutic role in atherosclerosis.

## Supporting information

S1 Fig MCC950 compound abrogates oxLDL-induced increase in pro-IL-1β protein expression. THP-1 cells were exposed to oxLDL, the NLRP3 inhibitor MCC950 (MCC) or their combination (MCC+OxL). Cell lysates were subjected to SDS-PAGE and immunoblotted with anti-pro-IL-1β (upper panel) or anti-vinculin antibody (bottom panel), as indicated. The band densities, relative to vinculin (loading control), were quantified using ImageJ (NIH, Bethesda, MD). Veh, vehicle. N = 3/group, * P<0.05 vs Veh, one-way ANOVA, Newman-Keuls test.
(TIF)

S2 Fig Olaparib inhibits OxLDL-mediated increase in IL-1β and IL-18 secretion. THP-1 cells were exposed to OxLDL, the PARP inhibitor olaparib (Olapa), or the NLRP3 inhibitor MCC950 (MCC). Secreted IL-1β (A) and IL-18 (B) in the cell culture medium were quantified by ELISA. N = 3/group, * P<0.05 vs vehicle, #P<0.05 vs OxLDL only, one-way ANOVA, Newman-Keuls test.
(TIF)

S3 Fig Olaparib attenuates the co-immunoprecipitation of IκBα with RelB or p52. Cell lysates were obtained from THP-1 monocytes exposed to reagents as indicated: Lane 1, vehicle; Lane 2, PARP inhibitor olaparib (5 mM, 24 hr); Lane 3, oxLDL (100mg/mL, 24 hr) or Lane 4, combination of oxLDL and olaparib. Protein complexes, co-immunoprecipitated with the indicated antibodies, were separated by SDS-PAGE and immunoblotted with the antibodies, as indicated. NR, normal rabbit IgG. Data shown are representative of three independent experiments. (**A**). Top panel was immunoblotted for RelB and bottom panel was immunoblotted for p52. Compared to vehicle (lane 1), OxLDL (lane 3), decreased co-immunoprecipitation of IκBαwith both RelB and p52 while olaparib (lane 4) reversed the effect of oxLDL. Olaparib alone (lane 2) had no effect on dissociation of IκBα with RelB and p52. **B**) Top panel was immunoblotted for IκBα. Bottom panel was immunoblotted for p52. Compared to vehicle lane (1), oxLDL alone (lane 3), decreased Co-IP of RelB with IκBα (top panel) but not with p52 (bottom panel). Further olaparib reversed the effect of oxLDL on the co-IP of RelB with IκBα (lane 4, olaparib+oxLDL). Olaparib alone (lane 2) had no effect.
(TIF)
