# Supplementary figures and images for: Poly-(ADP-ribose) polymerases inhibition by olaparib attenuates activities of the NLRP3 inflammasome and of NF-κB in THP-1 monocytes
Source: PLoS One. 2024 Feb 9;19(2):e0295837. doi: 10.1371/journal.pone.0295837 (PMC10857571; doi:10.1371/journal.pone.0295837)

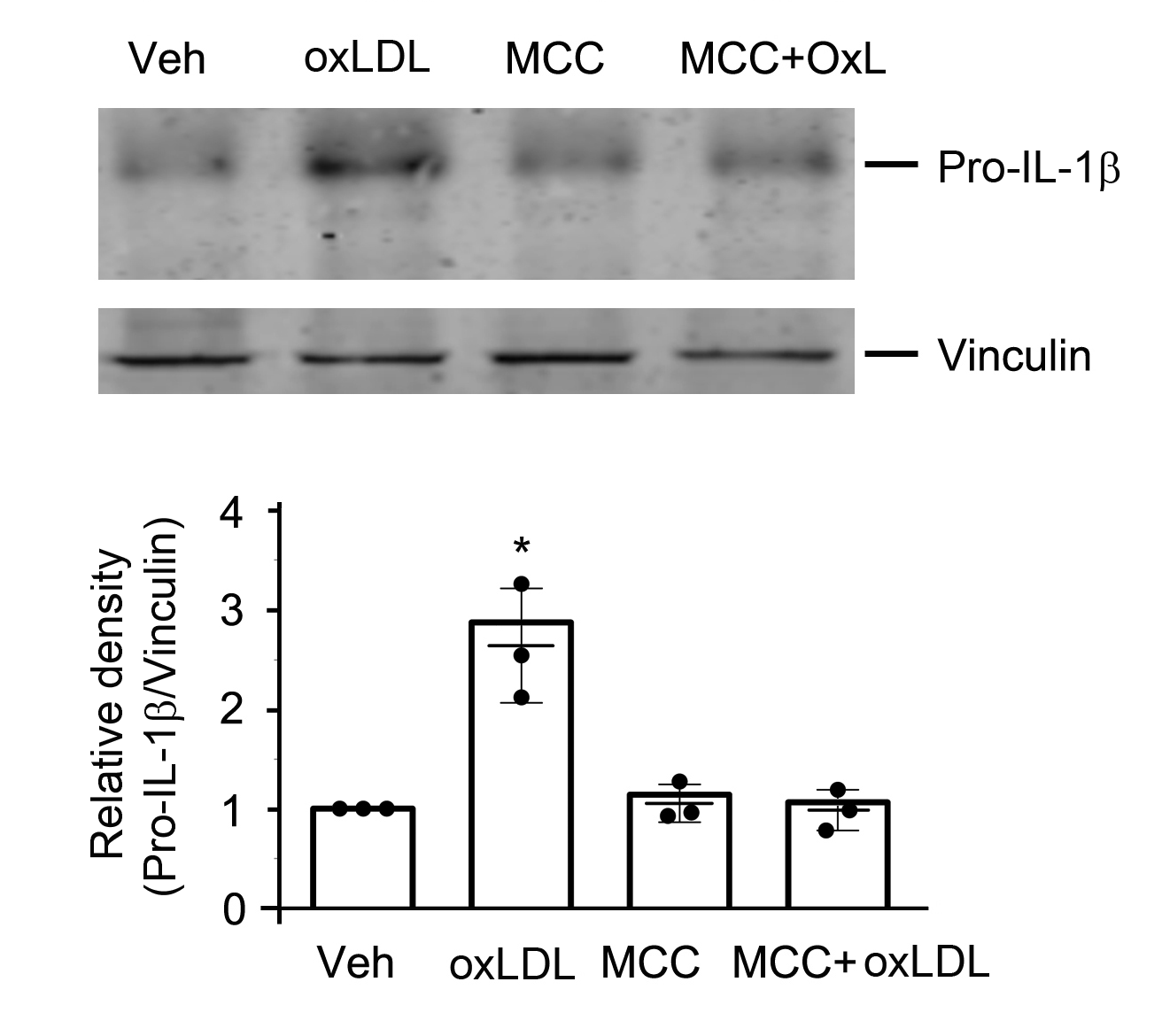

Supplement: S1 Fig — THP-1 cells were exposed to oxLDL, the NLRP3 inhibitor MCC950 (MCC) or their combination (MCC+OxL). Cell lysates were subjected to SDS-PAGE and immunoblotted with anti-pro-IL-1β (upper panel) or anti-vinculin antibody (bottom panel), as indicated. The band densities, relative to vinculin (loading control), were quantified using ImageJ (NIH, Bethesda, MD). Veh, vehicle. N = 3/group, * P<0.05 vs Veh, one-way ANOVA, Newman-Keuls test. (TIF) [file pone.0295837.s001.tif]

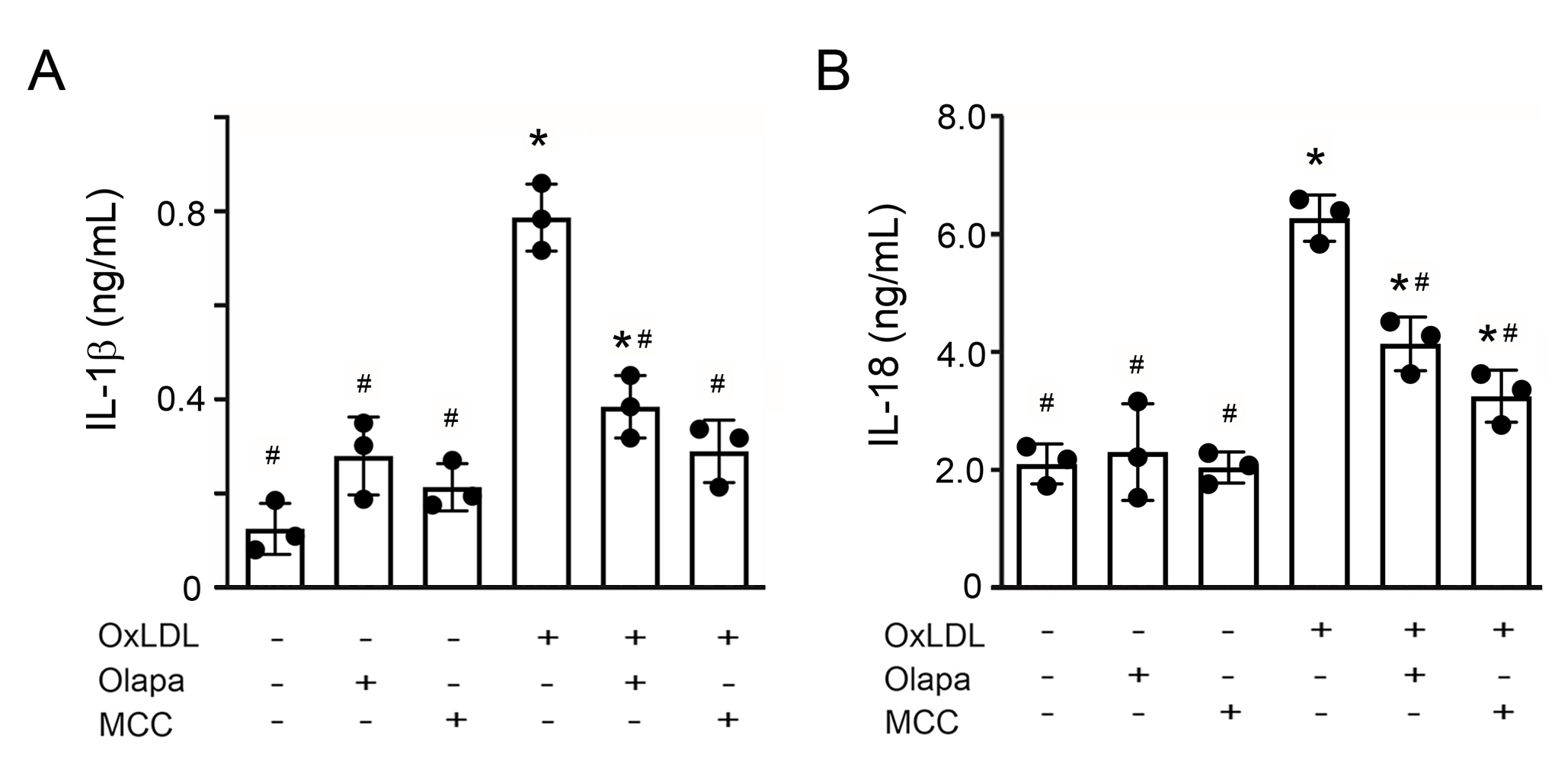

Supplement: S2 Fig — THP-1 cells were exposed to OxLDL, the PARP inhibitor olaparib (Olapa), or the NLRP3 inhibitor MCC950 (MCC). Secreted IL-1β (A) and IL-18 (B) in the cell culture medium were quantified by ELISA. N = 3/group, * P<0.05 vs vehicle, #P<0.05 vs OxLDL only, one-way ANOVA, Newman-Keuls test. (TIF) [file pone.0295837.s002.tif]

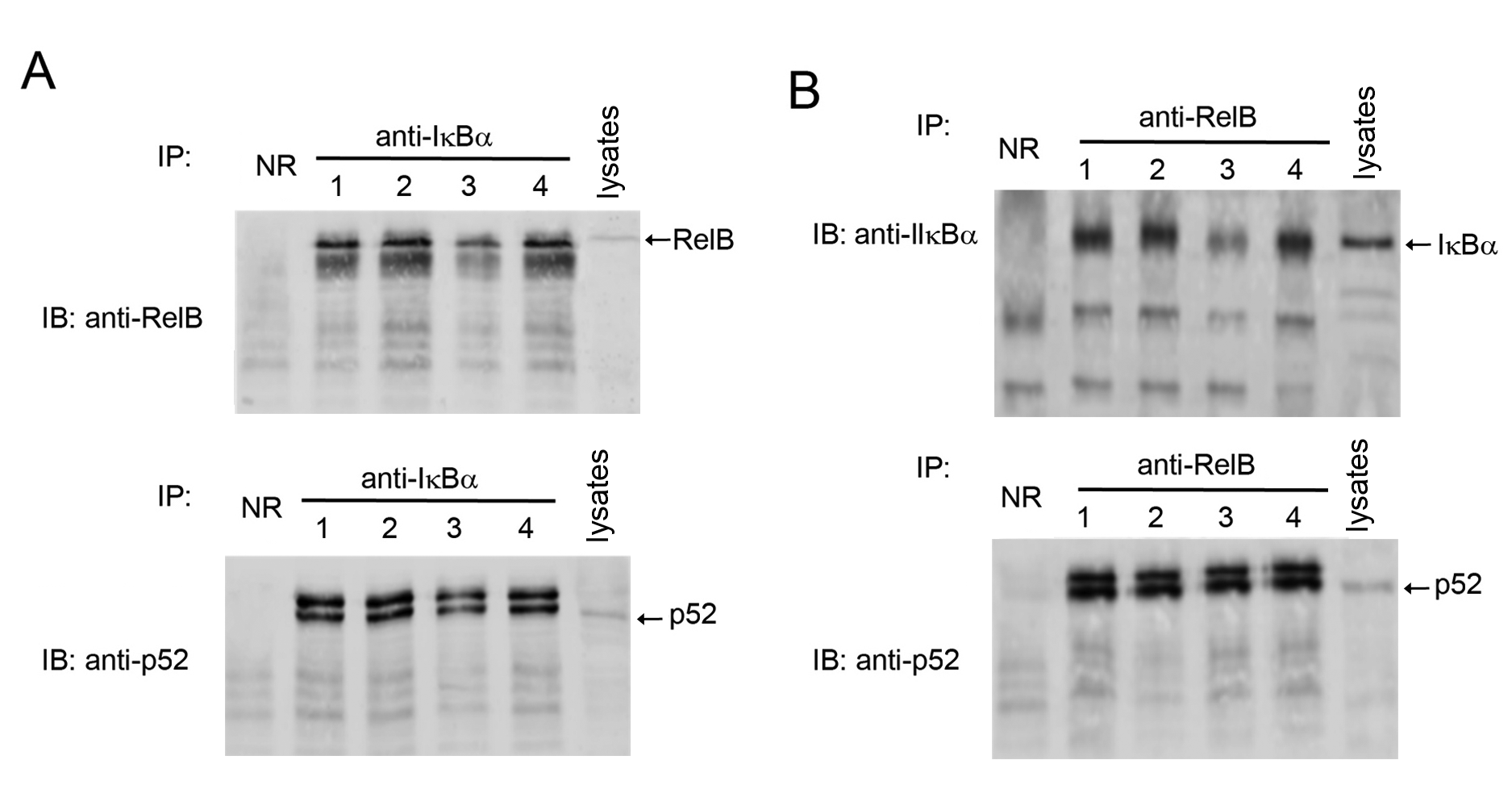

Supplement: S3 Fig — Cell lysates were obtained from THP-1 monocytes exposed to reagents as indicated: Lane 1, vehicle; Lane 2, PARP inhibitor olaparib (5 mM, 24 hr); Lane 3, oxLDL (100mg/mL, 24 hr) or Lane 4, combination of oxLDL and olaparib. Protein complexes, co-immunoprecipitated with the indicated antibodies, were separated by SDS-PAGE and immunoblotted with the antibodies, as indicated. NR, normal rabbit IgG. Data shown are representative of three independent experiments. (A). Top panel was immunoblotted for RelB and bottom panel was immunoblotted for p52. Compared to vehicle (lane 1), OxLDL (lane 3), decreased co-immunoprecipitation of IκBαwith both RelB and p52 while olaparib (lane 4) reversed the effect of oxLDL. Olaparib alone (lane 2) had no effect on dissociation of IκBα with RelB and p52. B) Top panel was immunoblotted for IκBα. Bottom panel was immunoblotted for p52. Compared to vehicle lane (1), oxLDL alone (lane 3), decreased Co-IP of RelB with IκBα (top panel) but not with p52 (bottom panel). Further olaparib reversed the effect of oxLDL on the co-IP of RelB with IκBα (lane 4, olaparib+oxLDL). Olaparib alone (lane 2) had no effect. (TIF) [file pone.0295837.s003.tif]
